# Analysis of Donor to Recipient Pathogen Transmission in Relation to Cold Ischemic Time and Other Selected Aspects of Lung Transplantation—Single Center Experience

**DOI:** 10.3390/pathogens12020306

**Published:** 2023-02-12

**Authors:** Maria Piotrowska, Małgorzata Edyta Wojtyś, Kajetan Kiełbowski, Michał Bielewicz, Piotr Wasilewski, Krzysztof Safranow, Tomasz Grodzki, Bartosz Kubisa

**Affiliations:** 1 Department of Thoracic Surgery and Transplantation, Pomeranian Medical University in Szczecin, Alfreda Sokołowskiego 11, 70-891 Szczecin, Poland; 2 Students’ Scientific Circle of the Department of Thoracic Surgery and Transplantation, Pomeranian Medical University in Szczecin, Alfreda Sokołowskiego 11, 70-891 Szczecin, Poland; 3 Department of Anesthesiology and Intensive Therapy, Independent Public Provincial Hospital Complex in Szczecin, Alfreda Sokołowskiego 11, 70-891 Szczecin, Poland; 4 Department of Biochemistry and Medical Chemistry, Pomeranian Medical University in Szczecin, Al. Powstańców Wielkopolskich 72, 70-111 Szczecin, Poland

**Keywords:** lung transplantation, donor to recipient transmission, pathogen colonization, cold ischemic time

## Abstract

Background: Infections are one of the leading causes of death in the early postoperative period after lung transplantation (LuTx). Methods: We analyzed 59 transplantations and culture results of the donor bronchial aspirates (DBA), graft endobronchial swabs (GES), and recipient cultures (RC) before and after the procedure (RBA). We correlated the results with a cold ischemic time (CIT), recipient intubation time, and length of stay in the hospital and intensive care unit (ICU), among others. Results: CIT of the first and second lungs were 403 and 541 min, respectively. Forty-two and eighty-three percent of cultures were positive in DBA and GES, respectively. Furthermore, positive results were obtained in 79.7% of RC and in 33.9% of RBA. Longer donor hospitalization was correlated with Gram-negative bacteria isolation in DBA. Longer CIT was associated with Gram-positive bacteria other than *Staphylococcus aureus* in GES and it resulted in longer recipient stay in the ICU. Furthermore, longer CIT resulted in the development of the new pathogens in RBA. Conclusion: Results of GES brought more clinically relevant information than DBA. Donor hospitalization was associated with the occurrence of Gram-negative bacteria. Positive cultures of DBA, GES, and RBA were not associated with recipient death.

## 1. Introduction

Lung transplantation (LuTx) is an accepted method of treatment in patients with end-stage respiratory diseases when conservative management is no longer beneficial [1]. Infections are one of the leading causes of death in the early post-transplant period. The risk of infection in LuTx comes from donor’s pathogens, nosocomial infection, or chronic colonization of the recipient’s airways. To exclude potential infectious complications in the direct postoperative period, collection of microbiological material immediately before the procedure is performed, together with antibiotic treatment of the recipient. Furthermore, one of the key factors in LuTx success is an appropriate cold ischemic time (CIT) which should not exceed 8 h [2]. The aim of this study was to evaluate the donor to recipient pathogen transmission and subsequent recipient infection with relation to CIT. We further analyzed the association between CIT and transmission with recipient prolonged intubation, length of stay in the intensive care unit (ICU), time of hospitalization, and efficacy of antibiotic therapy.

## 2. Materials and Methods

This is a retrospective analysis of the hospital records of patients undergoing LuTx from 21 December 2021 to 21 December 2018, at the Department of Thoracic Surgery and Transplantation of the Pomeranian Medical University in Szczecin and the medical data of donors from the POLTRANSPLANT database. The following data of recipients were analyzed: sex, age, cause of transplantation, type of the surgery, antibiotic therapy, intubation time and the need for reintubation within 7 days of surgery, length of stay in the ICU, total hospital stay, peri- and postoperative survival, and causes of death. Recipient infection was diagnosed based on clinical symptoms, laboratory tests, radiological imaging, bronchofiberoscopic examinations, and positive bronchial aspirate cultures. Perioperative death was defined as one that occurred during hospitalization following the transplantation procedure. Death due to infection was defined as a perioperative death with the symptoms of a recipient’s infection. All-cause death was any death throughout the entire study period. Donor sex, age, length of stay in the ICU (hours from admission until death), chest radiographs performed immediately before coordination, and CIT were analyzed. This retrospective study was based on reviewing medical databases without any additional interventions. In addition, consent from all patients was obtained. The bioethical commission of the Pomeranian Medical University evaluated the study and granted it an exemption status.

### 2.1. Microbiological Testing

Microbiological cultures were collected from four sites:

1. Donor bronchial aspirate (DBA)—during the final bronchoscopic organ assessment by the surgeon in the operating theater of the donor home hospital.
2. Graft endobronchial swab (GES, surgical material)—during its preparation before implantation in the recipient’s operating theater.
3. Recipient cultures (RC) before transplantation (nose and throat swabs, sputum, and urine)—after the recipient arrives at the hospital.
4. Recipient bronchial aspirate (RBA)—during the first and subsequent bronchofiberoscopies performed within 7 days after the transplant procedure.

The donor’s bronchial aspirates were collected during the bronchial tree assessment using an optical bronchofiberoscope (Olympus Europa) or a videobronchoscope (Karl Storz, Germany). The same devices were used for the examination of the recipients intubated or remaining in bed for other reasons. Subsequent bronchofiberoscopies of the recipient were performed in the Endoscopic Examination Laboratory using Pentax (Pentax Medical, Japan) or Olympus videobronchoscopes. The residual bronchial discharge was aspirated. Thereafter, 2 mL of physiological saline was administered intrabronchially, which was recovered into a sterile plastic transport tube. Graft endobronchial swabs and recipient nose and throat swabs were collected into sterile tubes with transport medium. Sputum and urine were transported in sterile plastic containers. The donor aspirates were delivered to the transplant center cooled in a transport refrigerator. The materials collected in the recipient’s center were sent to the microbiological laboratory at room temperature, immediately after collection. Microbiological diagnostics were carried out using the VITEK 2 automatic microbial identification and drug susceptibility system (BioMerieux SA.) Antibiotic susceptibility testing results were interpreted in accordance with the current recommendations of the European Committee for Susceptibility Testing (EUCAST).

### 2.2. Graft Preservation

After the organ procurement team arrived in the operating room of the donor hospital, bronchofiberoscopy was performed in order to exclude anatomical deviations, the presence of changes impeding bronchial patency, and to assess the amount and type of bronchial secretions with simultaneous collection for microbiological examinations. After excluding contraindications, the chest was opened. The lungs were rinsed with 4 L of preservative fluid cooled to 4 °C, which flowed through the left atrium into the pleura, from where it is removed. The block of both lungs was placed into a bowl, where each of the four openings of the pulmonary vein was backflushed with 250 mL of cold preservative fluid. The rinsed and cooled lungs were placed in a sterile bag with 1 L of cold preservative fluid. After the air was squeezed out and the first bag was tied, it was placed in the second bag with the addition of 1 L of cold saline. After releasing the air and tying, it was put into a third sterile bag, without the addition of cooling liquids. Then the organ was placed in a thermostable container cooled by four 1 L packages of frozen sterile water or in a container equipped with a compressor or thermoelectric system ensuring a stable temperature during transport. CIT was counted from the initiation of lavage with the preservative to the reperfusion after implantation.

### 2.3. Antimicrobial Therapy

As part of the prophylaxis based on the hospital formulary, recipients received meropenem (3 × 1 g), amikacin (15 mg/kg), and cloxacillin (4 × 1 g). Fluconazole (intravenous in 1st–3rd postoperative days), itraconazole (orally or through a probe), and voriconazole (orally and intravenous in CF patients or with history of *Aspergillus fumigatus* infection) were used as antifungal agents. Valganciclovir or ganciclovir were used as drugs active against cytomegalovirus. Topically, three agents were applied: amphotericin B (inhalation, 2 × 15 mg), nystatin (oral mucosa 4 × 1 mL suspension), and mupirocin (nasal vestibule twice a day). The basic set was used for a minimum of three days. It was modified depending on the recipient’s historical culture results or after obtaining the results of current microbiological tests. The first doses of antibiotics were administered intravenously in the operating theater before the beginning of the procedure. Antifungal drugs were introduced on the first postoperative day while antiviral agents were introduced within 10 days after the procedure.

### 2.4. Statistical Analysis

Measurable variables were presented using descriptive statistics including mean, standard deviation (SD), median, lower, and upper quartiles, and range (minimum-maximum). Most of the analyzed measurable variables showed distributions significantly different from the normal distribution (*p* < 0.05 for the Shapiro–Wilk test). Therefore, the non-parametric Mann–Whitney U test was used to compare the groups. Nominal variables were presented as counts and percentages, and Fisher’s exact two-tailed test was used to compare their distribution between groups. Survival was presented using the Kaplan–Meier curve. The strength of the relationship of the analyzed independent variables with the risk of death was calculated as the hazard ratio (HR) and its 95% confidence interval (95% CI) in the Cox proportional hazard model. *p* value < 0.05 was adopted as the threshold of statistical significance. Results were evaluated using the Statistica 13 program.

## 3. Results

### 3.1. Recipients

**Figure 1:**
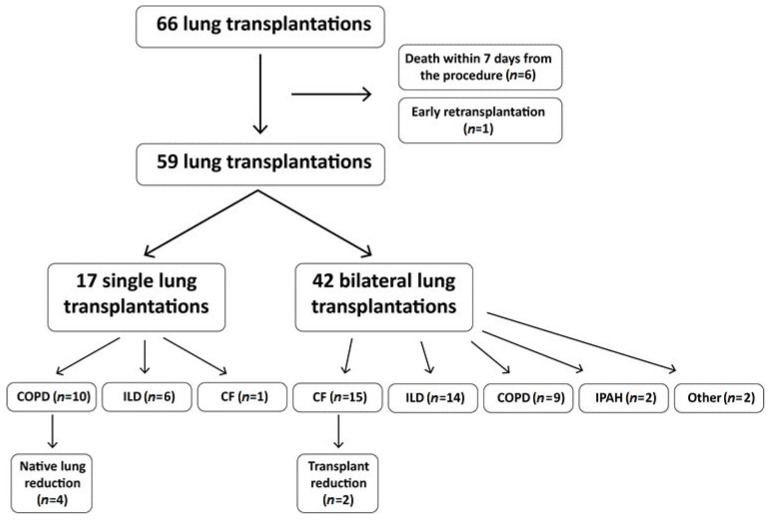
Characteristics of the study group—types of surgery and indications. CF—cystic fibrosis, COPD—chronic obstructive pulmonary disease, ILD—interstitial lung disease, IPAH—idiopathic pulmonary arterial hypertension.

In the study period, a total of 66 lung transplantations were performed in 65 patients. The data of 59 recipients (33 males—55.9% and 26 females—44.1%) who survived at least seven days after the procedure were further analyzed. Six excluded patients died due to surgical complications or cardiovascular events and were not associated with infections. One more excluded procedure was an early retransplantation on the 11th postoperative day. Indications for LuTx included interstitial lung disease (ILD; 20 patients—33.9%), followed by chronic obstructive pulmonary disease (COPD; 19 patients—32.2%), cystic fibrosis (CF; 16 patients—27.1%), with one late retransplantation, idiopathic pulmonary arterial hypertension (IPAH; 2 patients—3.4%), and other (2 patients—3.4%). Bilateral (BLT) and single lung transplantation (SLT) were performed in 42 (71.2%) and 17 (28.8%) cases, respectively. Six patients required lung parenchyma reduction due to native lung hyperinflation and chest asymmetry or short stature (Figure 1). The mean age of the whole group was 49 ± 13.3 years. Patients with CF represented the youngest group (31 ± 7.8 years versus 56 ± 6.8 years, *p* < 0.001). The mean age for ILD recipients was 57 ± 4.0 years and for COPD group 58 ± 4.9 years. SLT recipients were statistically older than BLT (59 ± 5.2 years versus 45 ± 13.6 years, *p* < 0.001). Due to the organizational reasons of the hospital, 13 recipients (22%) immediately after the procedure were treated in the intensive supervision room of the Department of Thoracic Surgery and Transplantation, and the remaining 46 (78%) were treated in the ICU where they stayed until the end of anesthetic procedures (intubation, dialysis). Prolonged intubation for at least three days or reintubation within seven days of surgery required 26 recipients (44%). The mean length of stay of 46 recipients in the ICU was 13 ± 15.1 days and the mean time of hospitalization was 33 ± 14.8 days (Table 1). In the perioperative period 12 patients (20.3%) died. As of 21 December 2019 (one year from the last transplantation included in the study), 33 patients remained alive. 

### 3.2. Donors

The 59 donors included 30 males (50.8%) and 29 females (49.2%), with a mean age of 39 years (±13.1). Radiological abnormalities were found in 23 donors (39%) (pneumothorax, vascular enhancement, areas of atelectasis, lung contusion, higher diaphragm dome), but they did not lead to the withdrawal of the organ. The average stay of donors in the ICU was 105 ± 77 h. The average CIT for the first or only lung was 403 ± 107 min, and for the second lung (42 procedures) it was 542 ± 114 min (Table 2).

### 3.3. Donor Bronchial Aspirates

**Figure 2:**
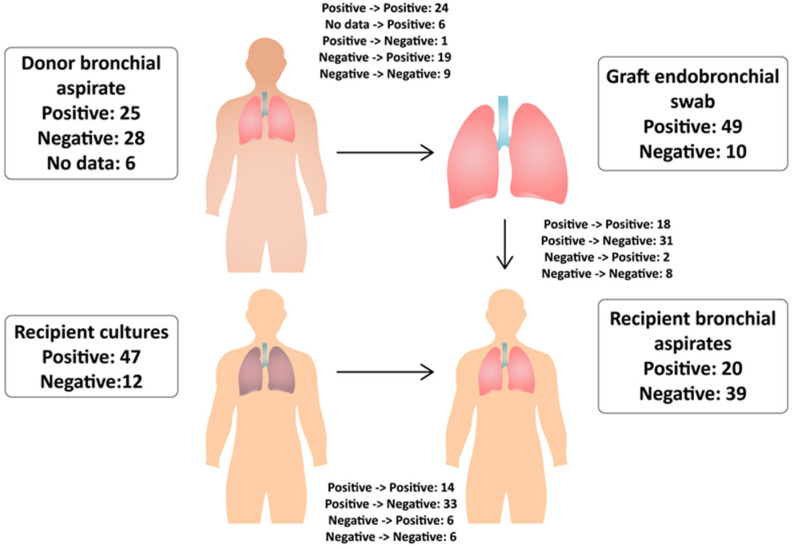
Number of positive and negative cultures and their transformation depending on the site of collection.

Cultures of bronchial aspirates of 53 donors were evaluated. The presence of pathogens was found in 25 cases (Figure 2). The cultures were dominated by Gram-negative bacteria which developed in 16 cases. Those pathogens were mainly *Klebsiella* (*n* = 5), including two strains of ESBL (+), followed by *Acinetobacter baumannii* (*n* = 4), Table 3. Gram-positive bacteria were grown in eight cases (methicillin-susceptible *Staphylococcus aureus* (MSSA)). In four cultures, two species of bacteria were found. *Candida* spp. were grown in seven cases, and they were the only species of fungi. There was no association between the donor length of stay in the ICU and positive cultures of donor bronchial aspirate compared to the negative cultures (mean 122 ± 63.2 h versus 98 ± 80.4 h, *p* > 0.05). However, such correlation has been observed in case of Gram-negative bacteria (132 ± 52.1 h for 16 cultures versus 100 ± 79.1 h for the remaining 37 cultures, *p* < 0.05). The longest ICU stay was associated with *A. baumannii* cultures (*n* = 4), as compared to the rest of the group (mean 176 ± 21.5 h versus 104 ± 73.2 h, *p* < 0.05). There was no relationship between the radiological findings reported for 23 donors and the positive results of the donor bronchial aspirates (*p* > 0.05). Positive donor aspirate cultures were associated with higher CRP values in the recipients on the 2nd, 3rd, 4th, and 7th postoperative days (16.41–8.35 mg/dL versus 10.47–4.71 mg/dL, *p* < 0.05). In case of Gram-negative bacteria, the same observation was made from 2nd to 7th postoperative days (18.84–6.55 mg/dL versus 11.01–2.72 mg/dL). Differences from days 4 to 6 were marked in *A. baumannii* cultures (13.71–10.93 mg/dL versus 5.76–3.09 mg/dL).

### 3.4. Graft Endobronchial Swab (Surgical Material)

Of the 59 graft endobronchial swabs collected during the preparation of the organ before implantation, 10 were negative (16.9%) and 49 were positive (83.1%). Compared to the results of donor bronchial aspirates, nine swabs remained negative and one previously positive became negative. Furthermore, pathogens were cultured out of 19 previously negative aspirates and in 24 previously positive aspirates. The same pathogens were cultured in 10 cases, additional cultures were seen in eight, while different results were obtained in six cases. Pathogens were also cultured in six cases where no data of preoperative donor bronchial aspirate were available. The surgical material was dominated by Gram-positive bacteria (27 cases) with 16 cases of *S. aureus* (including 1 MRSA) and other new species (Table 3). The number of positive mycological cultures has tripled into 22 cases. Gram-negative bacteria were grown in 23 cases, including seven strains of *Klebsiella* spp., *A. baumannii*, and five strains of *Escherichia coli*. Two *K. pneumoniae* strains produced ESBL and were previously found in the respective donor bronchial aspirates. Only one strain of *A. baumannii* with no donor bronchial aspirate data was fully susceptible to antibiotics (Table 4). There was no association between the donor’s length of stay in ICU and positive graft endobronchial swabs, as compared to the negative results (mean 108 ± 74.9 h versus 95 ± 57.5 h, *p* > 0.05). Statistical significance was demonstrated in seven cases in which *A. baumannii* was cultured (151 ± 36.6 h versus 99 ± 7.53 h, *p* < 0.05). Positive cultures of the graft endobronchial swab more often came from older donors (*p* < 0.05) with an average age of 41 ± 12.7 years compared to negative cultures from younger donors with 31 ± 12.4 years. Furthermore, the conversion from negative culture of bronchial aspirate collected in the donor’s operating theater to positive before implantation (*n* = 19) was statistically more frequent in the case of older donors (44 ± 11.0 years versus 36 ± 13.3 years, *p* < 0.05). The cultivation of Gram-positive bacteria other than *S. aureus* from surgical material (*n* = 13) was associated with longer CIT of the second lung, which was on average 600 ± 109 min for 12 grafts and 518 ± 108 min for the remaining 30 grafts (*p* < 0.05). Additionally, the presence of bacteria other than *S. aureus* was correlated with longer recipient ICU stay. Patients with such cultures (*n* = 11) spent approximately 21 ± 18.8 days in the ICU, as compared to patients with other cultures (*n* = 35, 10 ± 13.0 days). On the other hand, the cultivation of any Gram-negative bacteria from the surgical material (*n* = 23) was associated with a significantly longer stay of recipients in the hospital (38 ± 15.3 days versus 31 ± 14.0 days, *p* < 0.05). The simultaneous positive results of the culture of the donor bronchial aspirate and the surgical material (*n* = 24), regardless of the type of pathogen, were associated with higher CRP values in the recipient in the first postoperative week of observation (except for days 1and 5; 16.69–5.53 mg/dL versus 10.49–1.55 mg/dL, *p* < 0.05), mainly due to *Klebsiella* spp. and *A. baumannii* infections.

### 3.5. Recipient Cultures before Transplantation

Pathogens were found in the respiratory tract or urine immediately before transplantation in 47 recipients. Fungi colonization was observed in 32 cases. Gram-positive and Gram-negative bacteria were grown in 22 cases each. One recipient had two different species of Gram-positive bacteria, and five had two species of Gram-negative bacteria (Table 3). The most common findings were the presence of *S. aureus* in the respiratory tract and *P. aeruginosa* in the respiratory tract and urine. *Klebsiella* spp. was found in eight recipients (*K. pneumoniae* in seven, including four cases of ESBL (+) from urine, one *K. oxytoca* from the respiratory tract). *Acinetobacter baumannii* was not found in any recipient cultures before the procedure. The age of recipients infected with *P. aeruginosa* was significantly lower compared to the age of the rest of the study group (mean 35 ± 14.0 years versus 52 ± 11.4 years, *p* < 0.05). *Pseudomonas aeruginosa* strains that were cultured from recipients’ sputum and throats just before surgery, were only from seven CF patients and were more drug-resistant than strains obtained from the urine of two recipients (none with CF) or from two graft endobronchial swabs. Of the 14 cases where the recipient’s bronchial aspirate was still positive in the postoperative period, five showed the same pathogen, three of which showed a new species, while other species were found in nine cases. 

Four out of five patients who had grown the same pathogen in the postoperative period were qualified for LuTx due to CF. This observation was statistically significant compared to the other recipients with positive cultures collected immediately before transplantation (*n* = 47, *p* < 0.05). Recipients with positive cultures had significantly higher leukocytosis values on day 0 (12.7 ± 4.97 × 10^3^/µL versus 9.5 ± 2.9 × 10^3^/µL, *p* < 0.05). Recipients with the presence of the same pathogen immediately before transplantation and in the postoperative period, regardless of whether it was present alone or with another accompanying species, were characterized by significantly lower body temperature in the following days after the procedure (36.2–36.6 °C versus 36.8–37.4 °C, *p* < 0.05).

### 3.6. Recipient’s Bronchial Aspirates

Cultures performed within seven days of transplantation were negative in 39 recipients. Compared to the results of the graft endobronchial swabs, eight remained negative and 31 were negated. Out of 20 positive cultures, 10 showed the same and 10 a new pathogen. In two cases, the previous and new species were found while two previously negative cultures became positive (Figure 2). 

The recipient’s bronchial aspirates were dominated by Gram-negative bacteria, including five cases of A. baumannii XDR and P. aeruginosa each and two cases of K. pneumoniae. A. baumannii strains had the same profile of drug resistance as those identified in DBA/GES (Table 4). Strains of K. pneumoniae were MDR (multi-drug resistant) from DBA and GES and XDR cultured from recipients’ urine. Fungi were grown in nine cases (Table 3). The presence of the same species (*n* = 10) as in the previously positive graft endobronchial swab was more frequently found in older patients (mean age 52 ± 13.7 years versus 37 ± 12.2 years, *p* < 0.05). Furthermore, it was associated with statistically higher values of body temperature in the postoperative period, especially in cases of cultivation of the same and additional bacteria (38.0–38.7 °C versus 36.8–37.4 °C, *p* < 0.05). The appearance of a different pathogen (*n* = 10) than in the previously positive graft endobronchial swabs was more frequent in the group of younger recipients (mean 37 ± 11.7 years versus 54 ± 13.2 years, *p* < 0.05) and with higher CRP values just before the procedure (4.99 ± 6.76 mg/dL versus 0.56 ± 0.7 mg/dL, *p* < 0.05). Moreover, it was correlated with significantly higher values of postoperative leukocytosis on days 3 and 4 (21.3–26.3 × 10^3^/µL versus 12.2–14.2 × 10^3^/µL, *p* < 0.05). 

Compared to the whole group, the appearance of another species in the recipient’s bronchial aspirates (*n* = 10) had a significant relationship with the CIT of both the first/only lung (mean 476 ± 113 min versus 388 ± 100 min, *p* < 0.05) and the second lung (mean 613 ± 91 min versus 522 ± 113 min, *p* < 0.05). In addition, it was also noted that younger age and leukocytosis on days 3 to 5 were also correlated with additional aspirate species (mean age 37 ± 11.7 years versus 52 ± 12.3 years, *p* < 0.05; 16.2–26.3 × 10^3^/µL versus 11.9–17.99 × 10^3^/µL, respectively). The cultivation of the Gram-negative bacteria (*n* = 12) from the recipient’s bronchial aspirates was associated with higher CRP values on the 6th and 7th day after surgery (8.44-11.49 mg/dL versus 2,35–3.35 mg/dL, *p* < 0.05) and leukocytosis on the 5th and 6th days (15.63–16.62 × 10^3^/µL versus 11.88–12.79 × 10^3^/µL, *p* < 0.05). The presence of A. baumannii in the aspirate contributed to the higher CRP values in the following days of observation compared to the rest of patients (17.48–10.44 mg/dL versus 8,44–4.10 mg/dL, *p* < 0.05) with no significant differences in the leukocytosis value.

### 3.7. Postoperative Course

Prolonged intubation (more than three days) or reintubation within seven days from transplantation was required more often in younger recipients compared to the rest of the cohort. Longer intubation and reintubation were associated with longer CIT of both the first/only lung and the second lung, and resulted in a significantly longer recipient stay in the ICU (Table 5). 

There was no association between prolonged intubation or reintubation with the presence of pathogens in the donor bronchial aspirate, graft endobronchial swab, or recipient bronchial aspirates. Furthermore, no significant relationship was observed between the recipient’s length of stay in the ICU over seven days or the total hospitalization time over 30 days with positive culture results of the donor bronchial aspirates, a graft endobronchial swab, or the recipient bronchial aspirate. In the postoperative period, radiological examination revealed new inflammatory lesions in 40 recipients (67.8%). There was an association between those lesions and positive cultures of graft endobronchial swabs with a previously negative donor bronchial aspirate (*p* < 0.05). There was no correlation between the occurrence of radiological changes and positive recipient cultures before transplantation or the recipient’s bronchial aspirates. During the bronchofiberoscopic examination, purulent discharge in the bronchi was visualized in 37 recipients (62.7%)—12 (20.3%) in the first day after surgery and 32 (54.2%) on the following days. In seven patients, the discharge was found in both. The presence of purulent discharge in the first bronchofiberoscopic examination was significantly associated with positive culture results of recipient bronchial aspirates (*p* < 0.05). In this case, pathogens had already been cultured both in the recipient’s cultures before transplantation and in graft endobronchial swabs. Nevertheless, these findings were not correlated with the presence of significantly different values of inflammatory parameters (CRP and leukocytosis) and an increase in body temperature throughout the seven-day observation period.

In subsequent bronchofiberoscopic evaluations, there was no correlation between the presence of purulent discharge and a positive culture in the recipient bronchial aspirates. However, the presence of purulent discharge was associated with higher CRP values on the 3rd, 4th, 6th, and 7th postoperative days (9.48–4.74 mg/dL versus 8.35–1 mg/dL, *p* < 0.05), higher leukocytosis values on day 5 (13.95 × 10^3^/µL vs. 11.17 × 10^3^/µL, *p* < 0.05), and was associated with the appearance of new radiological findings in the recipient in the postoperative period (*p* < 0.01). 

**Figure 3:**
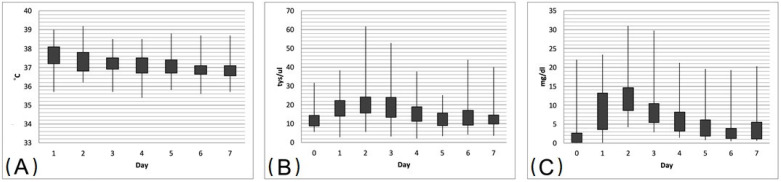
Analysis of postoperative course of the recipients. (**A**) Body temperature (range of values, quartile range) in the days following the procedure. (**B**) Leukocytosis (range of values, quartile range) in the days following immediately before and after the procedure (normal range 4.0–10.0 × 10^3^/µL). (**C**) C-reactive protein (range of values, quartile range) in the days following immediately before and after the procedure (normal range 0–0.5 mg/dL).

The highest values of the measured inflammatory parameters and temperature were achieved on the second day after the procedure with a tendency to a slight increase by the 6th and 7th postoperative days (Figure 3). Higher values of preoperative CRP and leukocytosis were observed in patients with CF compared to the rest of the study group (4.4 ± 5.76 mg/dL versus 1.31 ± 2.22 mg/dL, *p* < 0.05; 15.9 ± 6.24 × 10^3^/µL vs. 10.65 ± 3.17 × 10^3^/µL, *p* < 0.001, respectively). Patients with COPD had lower leukocytosis values (10.1 ± 2.98 × 10^3^/µL versus. 13.0 ± 5.22 × 10^3^/µL, *p* < 0.05) and CRP (0.45 mg/dL ± 0.53 versus 2.95 mg/dL ± 4.29, *p* < 0.001) before the procedure and no difference was found in the inflammatory parameters in the postoperative period.

### 3.8. Survival

**Figure 4:**
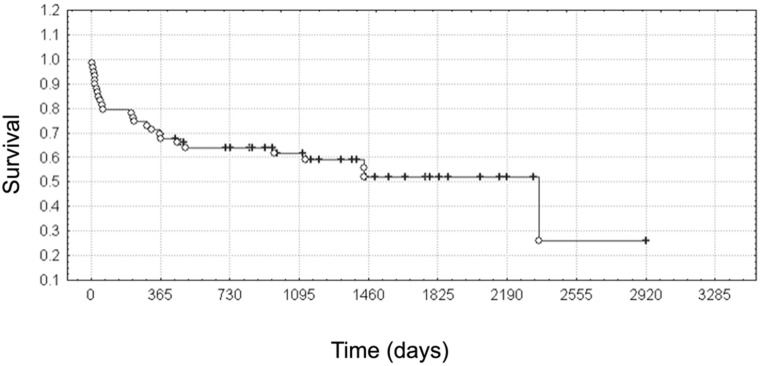
Kaplan–Meier survival curve. o—recipient’s death, +—end of observation on 21 December 2019.

Of the 59 patients, 12 died in the perioperative period. Deaths of four patients (33%) were due to infection caused by recipient, donor, or in-hospital pathogens. Three recipients (25%) died of cardiovascular disease and four patients (17%) died due to multi-organ failure and technical complications. Death of one recipient (8%) was caused by graft failure. After three months and one year after the procedure, 47 (79.7%) and 41 (69.5%) recipients survived, respectively. Until 21.12.2019 (the end of observation), 26 patients died while predicted five-year survival rate was 53% (Figure 4). We found no relationship between perioperative deaths or deaths due to infection and the presence of pathogens in any material collected from the donor or recipient. The analysis of the risk of death also did not show that positive cultures of donor bronchial aspirate, recipient bronchial aspirate, or recipient culture before transplantation increased this risk (Table 6). Perioperative deaths were related to the longer duration of the recipient’s stay in the ICU (26 ± 2.90 days versus 8 ± 9.7 days, *p* < 0.05). Similar observations were made in the case of deaths due to infection or any other reason (33 ± 24.7 days versus 11 ± 12.6 days, *p* < 0.05; 19 ± 19.3 days versus 7 ± 5.8 days, *p* < 0.05, respectively). Furthermore, perioperative death was correlated with a longer CIT of the second lung. Of the 12 deaths, 10 underwent BLT, and the mean second lung CIT was 607 ± 93 min, as compared to the remaining 32 patients who survived the perioperative period (521 ± 113 min, *p* < 0.05). There was no association between CIT of the second lung and death from infection. Perioperative deaths and deaths caused by infection were associated with higher CRP values on days 4, 6, and 7 (8.41–12.44 mg/dL versus 3.08–5.74 mg/dL, *p* < 0.05) and with lower CRP values on the 1st day in the case of perioperative deaths (5.98 mg/dL versus 9.53 mg/dL, *p* < 0.05) and overall deaths (7.27 mg/dL versus 10.38 mg/dL, *p* < 0.05). Perioperative deaths and deaths caused by infection occurred more often (*p* < 0.05) in recipients who had lower leukocytosis values immediately before transplantation (9.2 ± 3.34 × 10^3^/µL versus 12.8 ± 4.84 × 10^3^/µL, *p* < 0.05; 7.9 ± 1.77 × 10^3^/µL versus 12.4 ± 4.80 × 10^3^/µL, *p* < 0.05, respectively). There was no significant difference in postoperative leukocytosis and deaths. Of the four perioperative deaths caused by infection, only one recipient died from transmission of *A. baumannii* XDR from a donor. In another patient, despite the presence of *A. baumannii* in the graft endobronchial swab and the recipient bronchial aspirate, death occurred a long time after the procedure and was caused by a nosocomial infection with *P. aeruginosa*. *Pseudomonas aeruginosa* infection was also the cause of death for the other two recipients (nosocomial infection and pneumonia caused by its own MDR pathogenic flora).

### 3.9. Analysis of Antimicrobial Therapy

All recipients received at least two antibiotics from different groups. The basic set of carbapenem, aminoglycoside, and anti-staphylococcal penicillin was used in 35 (59.3%) recipients. The aminoglycoside was replaced with quinolone in six cases (10.2%) while treatment was started with two antimicrobial agents (no cloxacillin) in five recipients (8.5%). The remaining 13 recipients (22.0%) received initial treatment according to the antimicrobial susceptibility of pathogens as demonstrated by their own historical or donor cultures prior to coordination (combinations of piperacillin + tazobactam, teicoplanin, sulfamethoxazole + trimethoprim, linezolid, vancomycin, clindamycin, colistimethate sodium). In the group that did not receive the basic set, CF patients were the majority (14 recipients). Colistin was given mainly to CF patients (12 out of 16) and those infected with *A. baumannii*. Moreover, ampicillin + sulbactam was also used after confirmation of *A. baumannii* infection. In the prevention and treatment of fungal infections, all patients received topical nystatin and amphotericin B in inhalation (one exception). Triazole derivatives were introduced in 56 recipients. Echinocandins were given to 10 patients and, except for one case, were the second and third choice drugs, initiated after positive culture results. Prophylactic antiviral treatment was implemented in 27 recipients within seven days.

## 4. Discussion

Due to the risk of perioperative and postoperative complications related to the treatment and long-term results, the qualification process involves patients whose risk of death within two years without a transplant is greater than 50%. In addition, more than 80% probability of surviving for the next five years after the procedure are required, considering the proper function of the graft [3]. 

Mortality of patients after organ transplantation is the highest in the first months after surgery, and its causes change over time. Postoperative complications and infections are leading causes of deaths up to one year after LuTx, followed by chronic lung allograft dysfunction in subsequent years [4]. In this study, infections were the most common causes of death. However, patients who did not survive the first week after surgery due to surgical complications, cardiovascular events, and primary graft dysfunction (PGD) were not included in the study.

Postoperative infection can be a result of donor transmission, recipient’s flora, or nosocomial infection. The acceptance of the organ for transplantation should take place after the donor infection has been ruled out. Cultures of donor blood, bronchial aspirates, and preservative fluid were evaluated by several authors. Ruiz et al. included 197 procedures with 7.8%, 63.1%, and 29.1% of positive results, respectively [5]. Bunsow et al. reported 2.1%, 49%, and 23.3% of positive cultures accordingly [6]. Different outcomes were reported by Wainstain et al. with 12.7%, 83.6%, and 76.4% of positive cultures, respectively [7]. 

According to the cultures of donor bronchial aspirates and graft endobronchial swabs, positive swab cultures were found in 83% of cases, as compared to 47% of aspirates. The swabs showed almost four times more often Gram-positive flora, including doubled results of *S. aureus*, which resulted in equal proportions of Gram-positive and Gram-negative bacteria. The number of positive mycological cultures also increased. One of the reasons for such a significant difference may be contamination of the preservative fluid, which was shown by previously mentioned authors. However, examination of the preservative fluid and antibiotic therapy aimed at pathogens cultured from it are not well-established [8,9]. 

The second reason could be multiplication of the bacterial and fungal flora in the bronchial tree, depending on the time and temperature of the organ preservation. The use of a thermostable container and refrigerator allowed maintaining a constant temperature of 4–7 °C. Optimal temperature for development of psychrotrophic bacteria (*Pseudomonas* spp., *Acinetobacter* spp., *Staphylococcus* spp. among others) ranges from 20 to 30 °C but they can multiply in temperatures below 7 °C as well [10]. However, the conducted study did not show an association between the conversion from negative donor bronchial aspirates to positive bronchial transplant swab cultures and CIT. Another reason is the location where the material was collected. A swab of the graft was performed in the proximal sections of the bronchial tree (main bronchus and lobar orifice). In an intubated patient, the colonization of these places occurs faster than the colonization of the bronchioles and pulmonary alveoli rinsed during bronchial aspirate collection.

According to Chaney et al., CIT of lungs from an ideal donor should not exceed 4 h [11]. In studies evaluating the effect of CIT on recipient survival, bronchial complications, PGD, and acute or chronic rejection, 6 h were considered as borderline [12,13,14,15,16]. In an analysis performed by Thabut et al., CIT of the transplanted lungs longer than 6 h increased the recipient’s relative risk of death, which was the highest in the first year after surgery [16]. Another analysis of the International Society for Heart and Lung Transplantation (ISHLT) data from 2017 by Chambers et al. showed that exceeding 6 h of CIT was associated with worse 30-day and better 5-year survival rates. In addition, it was correlated with late development of bronchiolitis obliterans syndrome (BOS; the main type of chronic rejection) [13]. When analyzing the impact of CIT on survival, infection as a risk factor is not usually considered. Hennessy et al. evaluated the importance of geographical distance and CIT in LuTx and revealed that there was no association with BOS and one- and three-year survival rates. Nevertheless, a relationship with frequency of infections was noticed [17]. In the study by Ghaidan et al., infections were the most common causes of death (37%) in recipients with CIT that exceeded 6 h [18]. In contrast, Van Raemdonck et al. reported that exceeding 10 h also allows for good results of LuTx [19].

It has been demonstrated that the donor-recipient transmission of the infection negatively involves less than 1% of the organ transplant procedures. Nevertheless, it results in significant morbidity and mortality of recipients [20]. A study by Weinstein et al. did not show any transmission of infection associated with donor bacteremia or contamination of the preserving fluid, but 13% of lung recipients developed an infection associated with graft colonization, which was associated with inadequate antibiotic therapy [7]. Other studies concerning LuTx showed transmission of infection in 3–14% of positive donor bronchial aspirate cultures and a related mortality of 0–14% of infected recipients [5,6,7]. The transmission of pathogens in our analysis was much more frequent as it concerned 20% of positive cultures of the graft endobronchial swab, but only one recipient died from the infection. It seems that the transplanted lungs could be a source of infection in the recipient more often than other organs due to their function and anatomical structure which favor colonization. The secretion present in the distal bronchi might become a reservoir of pathogenic flora. One of the mechanisms of resistance to mechanical factors and antibiotics is the production of mucous biofilm [21]. 

Transmission of pathogens from the transplanted organ is usually the cause of infection in that organ. Ruiz I et al. demonstrated that out of 15 cases of transmission, four were pneumonia while tracheobronchitis was found in nine recipients [5]. The presence of purulent discharge in the recipient’s bronchi is a significant predictor to confirm an infection. Bronchofiberoscopy in the period immediately after transplantation is performed every 2–3 days to evacuate residual secretions, assess the healing of anastomosis, and collect material for microbiological tests, but visible inflammation of the mucosa is not sufficient to diagnose the infection. Epithelium of the graft mucosa after cold ischemia is exfoliated while submucosa is vivid and swollen. The healing process takes approximately three weeks. Tanaka et al. demonstrated the presence of purulent discharge in 89% of patients with pneumonia and in 25% of patients with tracheobronchitis [22]. In our study, we found purulent discharge in 62% of all recipients and its presence was significantly associated with radiographic changes in the chest (50% of patients). Despite such frequent detection of purulent discharge and radiological inflammatory changes, positive cultures of the recipient’s bronchial aspirate in the postoperative period were obtained only in 33% of recipients. However, radiological changes in lung recipients in the immediate postoperative period may arise not only due to infectious causes, but also in PGD and acute organ rejection. Radiological symptoms of PGD develop within 72 h of transplantation and are associated with specific gasometric disturbances. Acute and accelerated acute rejections are also observed within hours to a week after surgery.

This study included a seven-day observation period, assuming that this is the time of the highest risk of developing an infection depending on recipient or donor pathogens. Tanaka et al. demonstrated that 76% of cases of pneumonia in recipients occurred within one week after surgery [22]. Aguilar-Guisardo et al. report that almost half of pneumonia cases (40 out of 85) were diagnosed within the first month after LuTx. Furthermore, the diagnosis negatively influenced the one-year survival rate of recipients [23]. Riera et al. analyzed 24 cases of ventilator-associated pneumonia in 20 lung recipients. Early-onset pneumonia (EOP) was diagnosed in three patients while nine developed the disease one month after transplantation. Only in two cases of EOP were pathogens originating from the donor found (*S. aureus*). *Pseudomonas aeruginosa* was bred in 12 late-onset pneumonia cases, including eight multi-drug-resistant strains, which could indicate their in-hospital origin [24]. When considering the cause of respiratory infection in LuTx, one should take into account not only the transmission of an unknown pathogen from a mechanically ventilated donor, but also the multiplication of the recipient’s own pathogens. Zieliński et al. evaluated sputum, urine, and blood cultures from 200 patients waiting for LuTx. Among those, 31 were infected with alarm strains: 11 (35%) with *P. aeruginosa*, nine (29%) with *A. baumannii*, five (16%) with MRSA, and three (9%) with *K. pneumoniae* ESBL (+) without specifying the place of origin [25]. In this study different results were obtained. Among the positive recipient cultures performed before the procedure, *P. aeruginosa* was also the most common (19%), but no recipient was infected with MRSA or *A. baumannii*. *Klebsiella* spp, was grown in 17% of materials, including half of the strains ESBL (+).

Patients with CF are most at risk of developing infection from pathogens that inhabit their upper respiratory tract [26,27,28]. In the analysis of Gan et al., persistent infection was found in the post-transplant period in 60% (134 out of 224) of recipients with CF and in 6% (38 out of 612) of patients with another disease [29]. In the presented study, of 32 recipients with the presence of purulent discharge, nine suffered from CF. The dominant species in adult CF patients is *P. aeruginosa* (up to 80% of patients) [30]. A study by Beaume et al. showed that in patients with CF, the transplanted lungs already became colonized with *P. aeruginosa* on the 3rd day after the procedure and it was the main pathogen of the respiratory tract for the next three months despite the use of antibiotic therapy [31]. Another group of patients often colonized with *P. aeruginosa* are patients with bronchiectasis. Birch et al. found colonization in 45% of patients immediately before the lung transplant procedure. After surgery, colonization was still present in 21% of patients [32]. Despite the fact that *P. aeruginosa* infection is the most common etiological factor of bacterial pneumonia (24.6–60%) in lung transplant recipients [24,25], it seems that donor transmission is rarely the cause of *P. aeruginosa* infection, as it is usually an infection with one's own bacterial flora or a nosocomial infection associated with long-term treatment of the recipient in the ICU [5,6]. Our study supports these observations. 

*Klebsiella pneumoniae* can produce β-lactamases with an extended spectrum of activity (ESBL+) or carbapenemases (CPE), which contributed to an increased risk of morbidity and mortality in recent years [27,33,34]. Raviv et al. analyzed cultures of 136 lung recipients: *K. pneumoniae* ESBL (+) was found in 12 cases while *K. pneumoniae* CPE in 11 [35]. Despite the equally frequent presence of *K. pneumoniae* in the materials collected from the donor and recipient, their place of origin was different. Eight donor strains and only one recipient strain were grown from the material collected from the respiratory tract while the remaining seven recipient strains were derived from urine. This finding contrasted with the study by Tanaka et al., which showed that airways were colonized in 7% of recipients and in none of the donors [22]. Bunsow et al. demonstrated the presence of *K. pneumoniae* in 11 (4.6%) bronchial aspirates of donors, none was an ESBL (+) strain and only one was responsible for the recipient infection [6]. In the conducted study, transmission was found in one case, together with the infection with its own strain previously grown in the urine culture. None of the recipients died from *K. pneumoniae* infection. 

Another bacterial species that should be considered is *A. baumannii*. Despite the fact that it is a relatively pathogenic species, living mainly in water and soil, the literature draws attention to the multi-drug resistance of its strains related to prolonged hospitalization in the ICU and mechanical ventilation. The conducted study also confirmed the relationship between the donor’s stay in the ICU and the presence of *A. baumannii* in donor bronchial aspirates and graft endobronchial swabs. Additionally, six out of seven strains showed resistance to XDR-type drugs. It was much higher than in the study by Bunsow et al., where *A. baumannii* was only one of 118 different drug-resistant strains from lung donors and transmission of infection was not found [6]. In the analysis of Aguilar-Guisado et al., *A. baumannii* was the second (14%) most common etiological factor of pneumonia after LuTx [23]. Oh et al. reported that more than half of lung recipients (51/96) experienced infection with this pathogen and 19% died within 90 days of surgery [36]. In the presented study group, *A. baumannii* infection was the direct cause of death of one recipient. In three other cases, the infection was associated with the need for prolonged intubation and a stay in the hospital of more than 30 days.

Fazleen et al. did not find a relationship between CRP and prolonged intubation, ICU or hospital stay, and five-year survival rate after lung or heart and lung transplantation in the cohort of 72 CF patients [37]. In addition, multicenter analysis by Rello et al. failed to show a relationship between CRP and mortality after LuTx [38]. Our results contrasted with those findings. We observed higher CRP values in the postoperative period of recipients who received organs with positive cultures. Moreover, it was associated with the presence of purulent discharge in the bronchial tree and with *A. baumannii* infection after transplantation. There was also a correlation of CRP values on selected days with longer stay in the hospital and ICU, as well as a significant association with perioperative deaths and deaths due to infection. 

Antibiotic therapy is one of the basic elements of treatment in the postoperative period. In any case, there is a risk of multidrug-resistant strains resulting from the treatment of donors in the ICU and the chronic colonization of recipients, whose exacerbations require frequent antibiotic therapy, which favors the selection of strains insensitive to commonly used drugs. The antibiotic therapy should be composed of agents with a broad spectrum and different mechanisms of action. There are no unequivocal recommendations regarding the choice of the drug, but the range should include Gram-positive and Gram-negative bacteria, together with methicillin-resistant *Staphylococci* and *P. aeruginosa* [28,39,40]. In the conducted study, used regimens did not contain vancomycin or linezolid and only one case of a graft endobronchial swab was cultured with MRSA. The recipient developed pneumonia that resolved after treatment with linezolid. Introducing MRSA-targeted antibiotics only after donor cultures have been obtained seems to be a sufficient measure.

Presented study cannot be considered without certain limitations. Firstly, it was a retrospective, single-center analysis. Secondly, the study involved the beginning of our center’s activity, when the procedures were modified and fixed, which influenced the completeness of the data. Compared to the multicenter analyses, the study group was relatively small, and some events occurred too rarely to be considered statistically significant.

## 5. Conclusions

In conclusion, we have found that graft endobronchial swabs provide more useful information than donor bronchial aspirates. Additionally, we have shown that longer CIT of the second lung was associated with the presence of Gram-positive bacteria other than *Staphylococcus aureus* in the graft endobronchial swab. Cultivation of these bacteria was associated with longer recipient stay in the ICU. Furthermore, CIT of transplanted lungs was related to the need for reintubation or prolonged intubation and the length of recipient stay in the hospital. CIT of the second lung was associated with the length of stay in the ICU and perioperative death. In addition, longer duration of ICU stay for the donor favored the colonization of the respiratory tract with Gram-negative bacteria, especially *Acinetobacter baumannii*, which often showed multi-drug resistance and were not sensitive to prophylactic antibiotic therapy. Infections of the recipient with this pathogen were associated with significantly higher values of CRP. Ultimately, there was no correlation between *A. baumannii* infection and recipient stay in the ICU for more than seven days, hospitalization time of more than 30 days, or death due to infection. There was no relationship between the presence of pathogens in the donor bronchial aspirate, the graft endobronchial swab, and the recipient bronchial aspirates in the first week after transplantation with prolonged intubation, reintubation, prolonged recipient’s stay in the ICU for more than seven days or in the hospital for more than 30 days, and deaths. However, the presence of Gram-negative bacteria in the graft endobronchial swab contributed to the prolonged hospitalization of the recipient. CF patients chronically infected with *Pseudomonas aeruginosa* were the most frequently exposed to infection with their own pathogenic flora. Positive results of recipient bronchial aspirates preceded by the positive recipient culture prior to the surgery, or a graft endobronchial swab were associated with the presence of purulent discharge in the bronchi on the first, but not on the days following LuTx. Therefore, it proves the effectiveness of the antibiotic therapy. However, persistent positive bronchial aspirate cultures with positive preoperative recipient cultures were associated with increased incidence of deaths due to infection. 

## Figures and Tables

**Table 1:** Characteristics of recipients. ICU—intensive care unit.

	Population (*n* = 59)	Mean	SD	Median	Lower Quartile	Upper Quartile	Range	*p*-Value
Age (years)		49.66	13.33	57	39	59	20–65	<0.001
ICU stay * (days)	*n* = 46 78.0%	13.15	15.12	7	4.75	14.5	2–62	<0.001
Hospitalization (days)		33.97	14.83	31	24	41	7–73	<0.001
Survival on 21 December 2019	*n* = 33 55.9%	921.37	757.85	848	277	1445	7–2922	<0.01

* Due to organizational reasons of the hospital, 13 recipients were not treated in the ICU immediately after the procedure.

**Table 2:** Characteristics of donors. CIT 1—cold ischemic time of the first lung, CIT 2—cold ischemic time of the second lung (if transplanted), ICU—intensive care unit.

	Population (*n* = 59)	Mean	SD	Median	Lower Quartile	Upper Quartile	Range	*p*-Value
Age (years)		39.78	13.12	41	30	50	11–61	*p* = 0.08
ICU stay (hours)		105.92	77.02	109	42	142	8–334	*p* < 0.01
CIT 1 (min)	59/100%	403.27	107.02	400	330	480	150–630	*p* = 0.69
CIT 2 (min)	42/71.2%	541.74	114.14	540	477.5	600	270–740	*p* = 0.38

**Table 3:** Cultivated species of bacteria and fungi depending on the type of collected material. MLS—resistance to macrolides, lincosamides and streptogramin B, MRCNS—methicillin-resistant coagulase negative *Staphylococcus*, MRSA—methicillin-resistant *Staphylococcus aureus*, MRSE—methicillin-resistant *Staphylococcus epidermidis*, MSCNS—methicillin-susceptible coagulase-negative *Staphylococcus*, MSSA—methicillin-susceptible *Staphylococcus aureus*.

	Donor Bronchial Aspirate (*n* = 53)	Graft Endobronchial Swabs (*n* = 59)	Recipient Bronchial Aspirates (*n* = 59)	Recipient Cultures (*n* = 59)
**Gram-positive bacteria**				
*Staphylococcus aureus* MSSA	8	15	2	13
*Staphylococcus aureus* MRSA		1		
*Staphylococcus auricularis*		1		
*Staphylococcus haemolyticus* MRCNS, MLS		1	1	1
*Staphylococcus epidermidis* MRCNS, MRSE, MLS		4		4
*Staphylococcus lugudensis*		1		
*Staphylococcus hominis* MSCNS		1		
*Streptococcus anginosus*		1		
*Streptococcus agalactea* MLS				1
*Enterococcus faecalis*		2		3
*Enterococcus fecium*		1	1	
*Enterococcus casseliflavus*				1
*Corynebacterium ulcerans*		1		
**Gram-negative bacteria**				
*Enterobacter cloace*	2	4		4
*Klebsiella pneumoniae*	4	5	2	7
*Klebsiella oxytoca*	1	2		1
*Escherichia coli*	2	5		2
*Proteus mirabilis*		1	1	2
*Haemofilus influenzae*	1	1		
*Citrobacter freundii*	1	1		1
*Moraxella catharralis*				1
*Serratia odorifera*	1	1		
*Serratia marcescens*	1			
*Stenotrophomonas maltophilia*			1	
*Pseudomonas aeruginosa*		2	5	9
*Acinetobacter baumannii*	4	7	5	
*Achromobacter xylosoxidans*	1			
**Fungi**				
*Candida glabrata*	2	2	3	2
*Candida albicans*	5	17	6	29
*Candida crusei*		2		1
*Candida tropicalis*		1		
*Candida parapsylosis*				1
*Candida fumata*				1

**Table 4:** Drug susceptibility of *Acinetobacter baumannii* strains cultured from donor bronchial aspirate, recipient bronchial aspirates, and graft endobronchial swab. (-)—No data on donor aspirate, DBA—donor bronchial aspirate, GES—graft endobronchial swab, MIC—minimum inhibitory concentration, MS—medium-susceptible, R—resistant, RBA—recipient bronchial aspirate, S—susceptible, XDR—extended drug resistance, with the exception of sensitivity to at least one antibiotic of one or two classes. ^a^— Acinetobacter baumannii XDR.

Number of Strains	1	2 ^a^	3 ^a^	4 ^a^	5 ^a^	6 ^a^	7 ^a^
Material collection	(-)/ GES	DBA/ GES	DBA/ GES/ RBA	DBA/ GES/ RBA	DBA/ GES/ RBA	GES/ RBA	GES/ RBA
Drug sensitivity							
Piperacillin+ tazobactam		R	R		R	R	
Ticarcillin + clavulanic acid	S				R		
Ampicillin+ sulbactam	S	R	R	MIC 16	MS	R	S MS
Ceftazidime					R		
Cefepime	S	R	R	MS	R	MS	R
Cefotaxime		R					R
Ciprofloxacin	S	R	R	R	R	R	R
Levofloxacin			R			R	R
Imipenem	S	R	R	R	R	R	R
Meropenem		R	R	R	R	R	R
Gentamicin	S	R	R	R	S	S MS S	R
Tobramycin			R				R
Amikacin		R	R MS	S	R	R MS	R MS
Colistin		S	S	S	S	S	S
Tetracycline		R					
Trimethoprim + sulfamethoxazole	S	R	R	R	R	R	R

**Table 5:** Relationship between prolonged intubation and reintubation of the recipient with age, cold ischemic time, ICU stay, and hospitalization. CIT 1—cold ischemic time of the first/only lung, CIT 2—cold ischemic time of the second lung (if transplanted), ICU—intensive care unit. The results were calculated using Mann–Whitney U test. Due to organizational reasons of the hospital, 13 recipients were not treated in the ICU.

Parameter	Prolonged Intubation			Non-Prolonged Intubation			*p* Value
	*n*	Mean SD (Range)	Median (Range)	*n*	Mean SD (Range)	Median (Range)	
Age (years)	26	45 13.88 (21–65)	43 (33–58)	33	52 12.15 (20–64)	57 (50–59.75)	0.049
CIT 1 (min)	26	440 102.48 (245–630)	420 (360–519)	33	374.09 102.77 (150–570)	360 (303.75–435)	0.029
CIT 2 (min)	21	584 101.45 (380–740)	600 (495–660)	21	498 111.82 (277–690)	495 (412.5–580)	0.016
ICU stay (days)	22	21 18.36 (5–62)	13 (7.5–29.5)	24	5 3.20 (2–16)	5 (3–6)	<0.001
Hospitalization (days)	26	40 19.08 (7–73)	35 (73–23.5)	33	29 7.79 (16–53)	27 (24–32.75)	0.032

**Table 6:** Cox proportional hazard model with the risk of death depending on the positive results of the cultures. (-)—negative culture, (+)—positive culture, DBA—donor bronchial aspirate, GES—graft endobronchial swab, RBA—recipient bronchial aspirates, RC—recipient cultures (nose, throat, sputum, and urine).

	*p* Value	HR (95%CI)
DBA+	0.37	0.690 (0.309–1.539)
GES+	0.02 *	0.348 (0.144–0.842)
DBA-/GES+	0.03 *	0.322 (0.115–0.908)
RC+	0.84	0.911 (0.362–2.291)
RBA+	0.44	1.369 (0.619–3.028)

*—statistical significance.

## Data Availability

The donor data reported in this publication are contained in a database owned by POLTRANSPLANT and contain confidential elements. Therefore, it cannot be broadly disclosed or made publicly available. Recipient data supporting this study can be made available upon request to mariapio@o2.pl.
